# DNA Barcode Detects High Genetic Structure within Neotropical Bird Species

**DOI:** 10.1371/journal.pone.0028543

**Published:** 2011-12-07

**Authors:** Erika Sendra Tavares, Priscila Gonçalves, Cristina Yumi Miyaki, Allan J. Baker

**Affiliations:** 1 Department of Natural History, Royal Ontario Museum, Toronto, Ontario, Canada; 2 Departamento de Genética e Biologia Evolutiva, Instituto de Biociências, Universidade de São Paulo, São Paulo, São Paulo, Brazil; 3 Department of Zoology, University of Toronto, Toronto, Ontario, Canada; University of Veterinary Medicine Hanover, Germany

## Abstract

**Background:**

Towards lower latitudes the number of recognized species is not only higher, but also phylogeographic subdivision within species is more pronounced. Moreover, new genetically isolated populations are often described in recent phylogenies of Neotropical birds suggesting that the number of species in the region is underestimated. Previous *COI* barcoding of Argentinean bird species showed more complex patterns of regional divergence in the Neotropical than in the North American avifauna.

**Methods and Findings:**

Here we analyzed 1,431 samples from 561 different species to extend the Neotropical bird barcode survey to lower latitudes, and detected even higher geographic structure within species than reported previously. About 93% (520) of the species were identified correctly from their DNA barcodes. The remaining 41 species were not monophyletic in their *COI* sequences because they shared barcode sequences with closely related species (N = 21) or contained very divergent clusters suggestive of putative new species embedded within the gene tree (N = 20). Deep intraspecific divergences overlapping with among-species differences were detected in 48 species, often with samples from large geographic areas and several including multiple subspecies. This strong population genetic structure often coincided with breaks between different ecoregions or areas of endemism.

**Conclusions:**

The taxonomic uncertainty associated with the high incidence of non-monophyletic species and discovery of putative species obscures studies of historical patterns of species diversification in the Neotropical region. We showed that *COI* barcodes are a valuable tool to indicate which taxa would benefit from more extensive taxonomic revisions with multilocus approaches. Moreover, our results support hypotheses that the megadiversity of birds in the region is associated with multiple geographic processes starting well before the Quaternary and extending to more recent geological periods.

## Introduction

One of the striking patterns in geographic distribution of terrestrial biodiversity is the increase in species richness towards lower latitudes in several groups of organisms, including birds. The possible causes for this pattern is one of the highly debated topics in ecology and evolution, even though no definitive conclusion was yet been achieved [1], [2], [3], [4]. The Neotropical area alone holds a third of the recognized extant bird species (about 3,300 out of 10,000) [5], with a biodiversity hotspot in the tropical forests [6]. Moreover, recent phylogenies suggest the number of species in the area is underestimated because reproductively isolated lineages are frequently described in these studies [7], [8], [9], [10], [11]. In stark contrast to bird taxonomy in temperate zones, genetic evidence for species limits in the Neotropics is often discordant with traditional taxonomy due to the high incidence of species complexes. These complexes commonly feature gradual variation in morphological and behavioural characters, masking the occurrence of similar species that can be uncovered with genetic analyses [11], [12], [13], [14], [15].

DNA barcodes based on the 5′ portion of the cytochrome oxidase I gene (*COI*) linked with specimens vouchers and locality information provides a rapid and inexpensive method to identify species and detect ‘provisional new species’ [16]. Pilot DNA barcode surveys in birds of North America, sister-species pairs, and birds of Korea were successful in either identifying recognized species of birds, and detecting some potential new species, except for a minor proportion of cases where species are very recently diverged or hybridize [17], [18], [19], [20]. Critics questioned if the success observed in North American birds could be extrapolated to the tropics [21], where species clearly exhibit a higher level of phylogeographic subdivision [22]. However, DNA barcoding has subsequently proved to be highly successful in identifying Neotropical species of birds; all 16 species (100%) of antbirds (Thamnophilidae) that were barcoded [23] and 494 of 500 (95.8%) species of birds of Argentina [24] had distinguishable *COI* signatures. The screening of Argentinean birds also detected 21 species with deep intraspecific structure, and revealed more complex patterns of regional divergence in the Neotropical than in the North American avifauna [24]. Even though more species will doubtlessly be shown to share barcodes when complete coverage of species and genera is available, it is clear that large-scale sequencing of *COI* associated with vouchered specimens and locality information is a valuable tool in understanding genetic differentiation within and among species of birds [17], [21], [24], [25].

In this study we increased the coverage of Neotropical bird species that have been barcoded by adding 637 samples from 431 species, with higher representation in tropical forest areas of Brazil and Guyana, but also including samples from localities ranging from Mexico to Argentina and Chile. We compared these sequences with previously published sequences of congeneric species of Neotropical birds, totaling 1,431 samples from 561 different species of birds, 296 of which were represented by multiple individuals. We showed that a high success rate in species identification (93%) with DNA barcodes can be achieved in this large sample of avian biodiversity from the mega-diverse Neotropical region similar to that obtained in broad geographic surveys in the Nearctic and Palearctic regions of the world. Additionally, a higher percentage (12%) of species had multiple deep phylogeographic splits than in previous surveys, some of which are likely reproductively isolated lineages.

## Results

### Species identification in Neotropical birds

**Figure 1:**
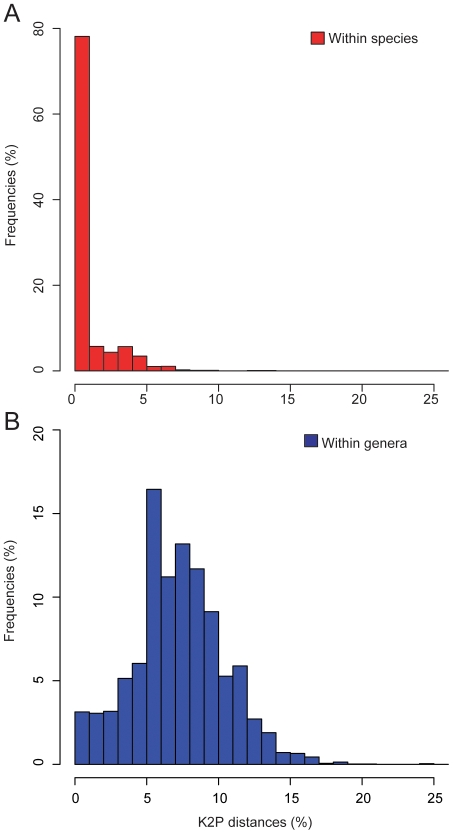
Frequency distribution of K2P distances within and among species of Neotropical birds. A) Pairwise distance comparisons within currently recognized species. B) Pairwise distance comparisons among congeners, excluding withinspecies comparisons.

About 93% of the species in our sample (520 out of 561) did not share sequences with any other species included in the analysis, and when multiple individuals were sampled (296 species), mean genetic distances among individuals were lower than to the closest species from the same genus (File S1, Table S1). Kimura 2-Parameter genetic distances (K2P) within-species had a wide range (0 up to 13.7%), with more than 75% of the observations below 1% K2P. Conversely, 10% of the pairwise comparisons were higher than 3% K2P (range 3.1–13.7%), overlapping considerably with among-species variation (Figure 1, Table S1). Pair-wise comparisons among-species of the same genus were distributed from 0.08 to 20.3% K2P with most of the comparisons observed between 5–15% K2P (Figure 1). Extremely high genetic distances suggestive of higher rates of evolution or ancient divergences were observed among species within *Trogon* (Trogoniformes) and *Crypturellus* (Tinamiformes), with maximum distances of 19, and 20.3%, respectively. One specimen identified as *Nothoprocta ornata* differed from other species in the genus by 24.7%, but was only 0.34% divergent from specimens of **Tinamotis pentlandii.** Hence it was either incorrectly identified in the field or possibly is a hybrid between the two genera, as both species occur near to the collecting locality in Chile.

A total of 41 species did not have unique barcodes, of which 21 share sequences with other species (Table 1). Eight of those species despite being reciprocally monophyletic, or represented by one sample, only differed from their sister species by 1 to 6 diagnostic characters, or 0.14 to 0.86% K2P distance. Aggregation of closely related haplotypes in phylogenetic trees can either represent distinct taxonomic units, or random branches of lineages within the same taxonomic group [26]. To distinguish between the two scenarios, we applied a statistical test of taxonomic distinctiveness proposed by Rosenberg [26] for sister species differing by less than 1% K2P. With the current limited sampling of individuals, chance occurrence of reciprocal monophyly between species could not be rejected (p>0.05), so they were considered not distinguishable by *COI* barcodes (Table 1). Species pairs differed by few nucleotide substitutions, with marginal values for the test of chance occurrence of reciprocal monophyly (0.01<p<0.05). Thus the following species groups were considered to be distinguishable by *COI* barcodes: the ducks *Anas puna*/*versicolor* (p = 0.01) [24], the greenfinches *Carduelis atrata/barbata/versicolor* (p = 0.01) [24] and the orioles *Icterus cayanensis*/*chrysocephalus* (p = 0.03).

**Table 1:** Species without unique DNA barcodes.

Species	Sampling (#)	Category^a^	Share barcodes or is very closely related to:	Test of chance reciprocal monophyly (p)^c^
*Anas flavirostris*	7	I and IV	*Anas sibilatrix*	-
*Anas sibilatrix*	6	I	*Anas flavirostris*	-
*Basileuterus culicivorus*	4	III	*Basileuterus hypoleucus*	0.1
*Basileuterus hypoleucus*	1	III	*Basileuterus culicivorus*	0.1
*Celeus elegans*	1	III	*Celeus lugubris*	0.17
*Celeus lugubris*	3	III	*Celeus elegans*	0.17
*Conopophaga lineata*	4	IV	-	-
*Dendrocincla fuliginosa*	4	IV	-	-
*Gymnopithys rufigula*	3	II	*Gymnopithys leucaspis*	-
*Gymnopithys leucaspis*	1	II	*Gymnopithys rufigula*	-
*Hemitriccus minor*	2	IV	-	-
*Herpsilochmus atricapillus*	1	III	*Herpsilochmus sellowi*	-
*Herpsilochmus sellowi*	1	III	*Herpsilochmus atricapillus*	-
*Hylophilus ochraceiceps*	3	I and IV	*Hylophilus semicinereus*	*-*
*Hylophilus semicinereus*	1	I	*Hylophilus ochraceiceps*	*-*
*Myiarchus swainsoni*	3	IV	-	-
*Myiobius barbatus*	5	IV	-	-
*Penelope jacquacu*	2	IV	-	-
*Phaethornis superciliosus*	4	IV	-	-
*Picumnus temminckii*	3	II	*Picumnus pygmaeus*	*-*
*Picumnus pygmaeus*	1	II	*Picumnus temminckii*	*-*
*Sporophila bouvreuil*	1	I	six *Sporophila* species	-
*Sporophila cinnamomea* b	2	I	six *Sporophila* species	-
*Sporophila hypochroma* b	1	I	six *Sporophila* species	-
*Sporophila hypoxantha* b	4	I	six *Sporophila* species	-
*Sporophila palustris* b	2	I	six *Sporophila* species	-
*Sporophila ruficollis* b	2	I	six *Sporophila* species	-
*Sporophila zelichi* b	1	I	six *Sporophila* species	-
*Synallaxis gujanensis*	1	I	*Synallaxis rutilans*	*-*
*Synallaxis rutilans*	4	I and IV	*Synallaxis gujanensis*	*-*
*Tachyeres patagonicus*	1	I	*Tachyeres pteneres*	*-*
*Tachyeres pteneres*	2	I	*Tachyeres patagonicus*	*-*
*Thraupis palmarum*	1	III	*Thraupis sayaca*	0.05
*Thraupis sayaca*	6	III	*Thraupis palmarum*	0.05
*Tolmomyias assimilis*	3	IV	-	-
*Turdus albicolis*	4	I and IV	*Turdus leucomelas*	*-*
*Turdus leucomelas*	9	I and IV	*Turdus albicolis*	*-*
*Tyrannus melancholicus*	7	IV	-	-
*Veniliornis frontalis* b	1	IV	*Veniliornis passerinus*	
*Veniliornis passerinus* b	4	IV	*Veniliornis frontalis*	
*Vireo olivaceus*	10	IV	-	-

a I) share barcodes with sympatric species; II) share barcodes with allopatric species; III) monophyletic but very closely related to sister species; IV) paraphyletic species with lineages more than 1.5% divergent (see Table 2).

b Previouly reported by Kerr et al [24] and/or Campagna et al [62].

c Only performed for reciprocally monophyletic species pairs.

**Figure 2:**
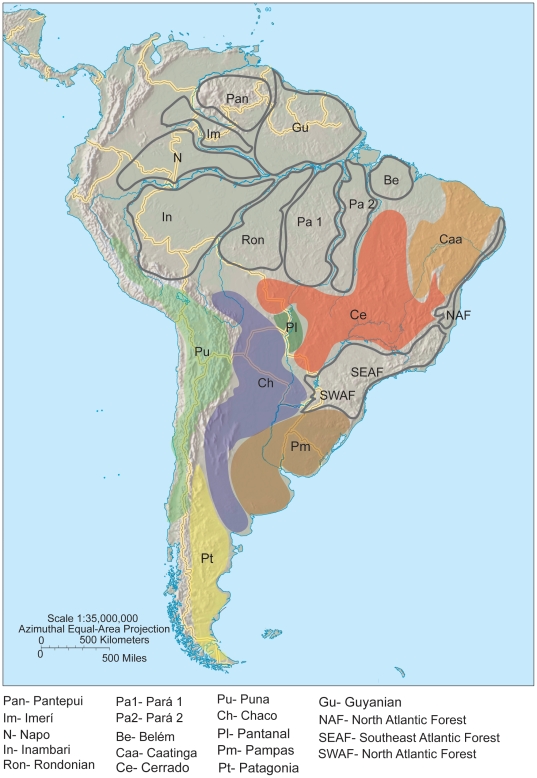
Areas of endemism and ecoregions of Neotropical bird specimens studied. Map of South America showing areas of endemism [38], [61] and ecoregions [5] used in the present study to group sample localities.

**Figure 3:**
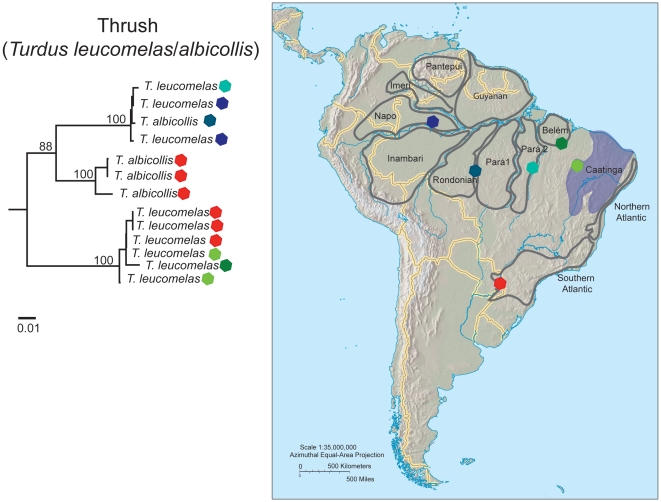
Maximum likelihood tree topology of the pale-breasted (*Turdus leucomelas*) and white-necked (*Turdus albicollis*) thrushes based on DNA barcodes. Scale bar shows the number of substitutions/site. Bootstrap values higher than 70% (100 replicates) are indicated on the corresponding branches. Colour-coded sample localities are represented on the map.

Sixteen species had multiple divergent clusters (K2P genetic distances between 1.54 up to 13.7%), not recovered monophyletic with COI, that often corresponded to samples from different areas of endemism or ecoregions (Table 1- cat. IV, Table 2, Figure 2). A few exceptions were observed, where paraphyletic divergent specimens were found in the same geographic locality. For instance, specimens from the long-tailed hermit (*Phaethornis superciliosus*) from Aripuanã and Juruena, both within the Rondonian area of endemism, were 8% divergent, and the specimen from Juruena differed from a scale-throated hermit (*P. eurynome*) from Southern Atlantic forest by 7.4%. Even more strikingly, two samples of the yellow-margined flycatcher (*Tolmomyias assimilis*) from Napo were 8.3% divergent. The species pair of thrushes *Turdus albicollis/leucomelas* were paraphyletic in their *COI* sequences, sharing barcodes in their Amazonian distribution (Figure 3).

**Table 2:** Species recovered as paraphyletic with COI barcode (Table 1, IV, File S1).

Species	Cluster locality (sampling)^a^	Range K2P (% within clusters)^b^	Maximum K2P (% among clusters)^b^
*Anas flavirostris*	a. Pt/Ch (2)	0	a vs b = 6.3
	b. Pu/Pt (4)	0.15	
*Conopophaga lineata*	a. NAF (1)	-	a vs b = 9.64
	b. SWAF/SEAF (3)	0.30–1.44	-
*Dendrocincla fuliginosa*	a. N (1)	-	a vs b = 5.58
	b. Gu/Be (3)	0–1	
*Hylophilus ochraceiceps*	a. Im/N (2)	0.14	a vs b = 7.07
	b. Ron/Pa1 (1)	-	
*Myiarchus swainsoni*	a. SEAF (1)	0.59	a vs b = 3.83
	b. Pt (2)	-	-
*Myiobius barbatus*	a. Be (1)	-	a vs b = 13.71
	b. Pa2 (1)	-	a vs c = 13.43
	c. SWAF/SEAF (2)	0.28–0.47	b vs c = 12.67
*Penelope jacquacu*	a. N (1)	-	a vs b = 4.5
	b. In (2)	0	-
*Phaethornis superciliosus*	a. Im (1)	-	a vs b = 7.61
	b. Ron/Pa2 (2)	0.15	a vs c = 8.61
	c. Ron/Pa1 (1)	-	b vs c = 7.42
*Synallaxis rutilans*	a. Be/Im (2)	0.7	a vs b = 1.54
	b. Ron/Pa1 (2)	0	-
*Tolmomyias assimilis*	a. N (1)	-	a vs b = 8.30
	b. Ron (1)	-	a vs c = 8.47
	c. N (1)	-	b vs c = 7.09
*Turdus albicolis*	a. Ron/Pa1 (1)	-	a vs b = 4.51
	b. SWAF (3)	0	-
*Turdus leucomelas*	a. Caa/Be/SWAF (6)	0–0.58	a vs b = 6.14
	b. N/Pa2 (2)	0–0.15	
*Tyrannus melancholicus*	a. SEAF (1)	-	
	b. Caa/Pu/Ch (6)	0	a vs b = 2.10
*Veniliornis passerinus*	a. Caa (1)	-	a vs b = 1.95
	b. Ch (3)	0	
*Vireo olivaceus*	a. N (2)	0	a vs b = 2.94
	b. Pu/Ch (3)	0.3–0.43	a vs c = 3.28
	c. SWAF/SEAF/Ch (5)	0–0.16	b vs c = 3.09

a Area of endemism or ecoregion listed for each cluster.

b range and maximum K2P genetic distances within each cluster, and among clusters, respectively. Geographic areas coded according to Figure 2.

### Deep genetic structure within Neotropical bird species

**Figure 4:**
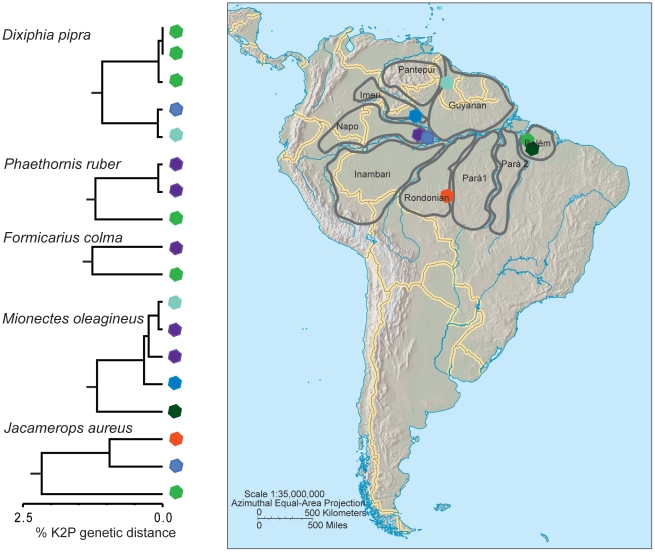
Species with genetic discontinuities between the Napo and Belem areas of endemism. Colour-coded sample localities are represented on the map.

**Figure 5:**
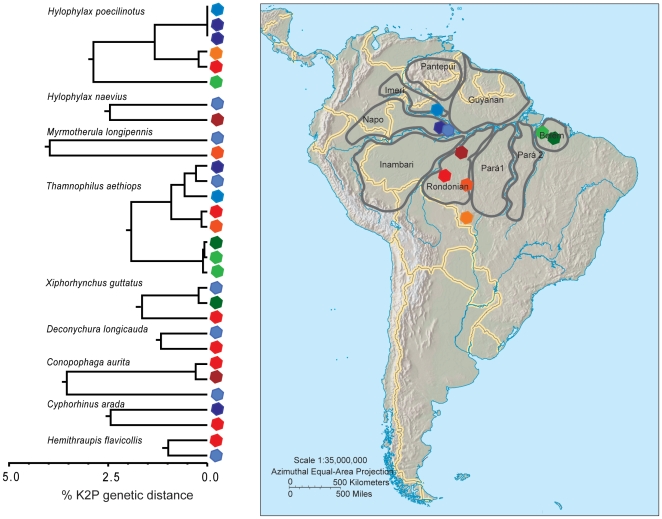
Species with genetic discontinuities among the areas of endemism Napo, Rondonian and Belém. Colour-coded sample localities are represented on the map.

Deep intraspecific divergences in 48 species overlapped widely with among-species distances (K2P 1.6 to 7.8%, Table 3). These genetically structured species belong to 21 bird families from nine different bird orders, most frequently represented by antwrens (Thamnophilidae, Passeriformes). Most of the species with deep genetic structure were broadly distributed in the Neotropics, and several are subdivided into multiple subspecies [27]. Often samples from different areas of endemism or different ecoregions were the most divergent within species (Table 3). Some species showed genetic discontinuities in some pairs of geographic areas, but not in others, such as the ochre-bellied flycatcher (*Mionectes oleagineus*). Samples from the Napo, Imeri and Guyanian areas of endemism were not very distinct genetically, but specimens from Belém were 2.76% divergent from the others (Figure 4). All samples of the white-shouldered antshrike (*Thamnophilus aethiops*) from different areas of endemism (Belém, Rondonian, Imeri, and Napo) had deep instraspecific genetic variation. The deepest split was between Belém and the other areas, and then the next split was between Rondonian, Imeri, and Napo (Figure 5).

**Table 3:** Species with deep within species divergences recovered monophyletic with COI barcodes.

Species	Cluster or sample locality (sampling)^a^	Max K2PD (%)^b^
*Arremon taciturnus*	a. NAF (1) b. Ron/Pa1 (1) c. Be (1)	1.6
*Chloroceryle aenea*	a. SEAF (1) b. Ron (1)	2.21
*Cnemotriccus fuscatus*	a. Ron/Pa1 (1) b. Caa (1)	5.97
*Conopophaga aurita*	a. Ron (2) b. N (1)	7.48
*Crypturellus tataupa*	a. Caa (1) b. SWAF (1)	3.94
*Cyanocompsa brissonii*	a. Ch (4) Pu (2) b. Ch (2)/Caa (1)	2.66
*Cyclarhis gujanensis*	a. Caa (1) b. Ch (3)/Pu (1)	3.04
*Cyphorhinus arada*	a. Ron (1) b. N (1)	4.86
*Dacnis cayana*	a. N (1) b. SEAF (1)	2.92
*Deconychura longicauda*	a. Ron (1) b. N (1)	2.33
*Deconychura stictolaema*	a. Ron/Pa1 (1) b. N	2.28
*Dixiphia pipra*	a. Be (3) b. N (1)/Gu (1)	2.52
*Formicarius colma*	a. N (1) b. Be (1)	2.5
*Formicivora grisea*	a. Ron/Pa1 (1) b. NAF (1)	1.64
*Galbula dea*	a. Gu (1) b. Be (1)	5.14
*Geothlypis aequinoctialis*	a. Ch (4) b. Pu (1)	2.36
*Glyphorynchus spirurus*	a. Ron/Pa1 (1) b. N (3)	6.02
*Hemithraupis flavicollis*	a. Ron (1) b. N (1)	1.97
*Hylophylax naevius*	a. Ron (1) b. N (1)	4.87
*Hylophylax poecilinotus*	a. N (2)/Im (1) b. Ron/Pa1 (2)	6.05
*Hypocnemis cantator*	a. Gu (1) b. Ro (1)	4.05
*Jacamerops aureus*	a. Ron (1) b. N (1) c. Be (1)	4.49
*Malacoptila rufa*	a. Ron/Pa1 (3) b. Be (1)	3.95
*Micrastur gilvicollis*	a. Ron/Pa1 (1) b. In (2)	5.95
*Microcerculus marginatus*	a. Ron (1) b. Be (1)	1.61
*Mionectes oleagineus*	a. Be (1) b. N (2)/Im (1)/Gu(2)	2.76
*Monasa morphoeus*	a. N (2) b. Ron/Pa1 (3)	1.67
*Myiobius atricaudus*	a. Caa (1) b. SEAF (1)	1.74
*Myrmeciza atrothorax*	a. Ron/Pa1 (1) b. Im (1)	3.31
*Myrmoborus myotherinus*	a. N (1) b. Ron/Pa1 (1)	2.83
*Myrmotherula hauxwelli*	a. Be (1) b. Ron (1)	3.43
*Myrmotherula longipennis*	a. Ron (1) b. N (1)	7.83
*Nyctidromus albicollis*	a. N (1)/Gu(1) b. Ch(1)	2.66
*Phaeomyias murina*	a. Caa (1) b. Pu (1)	3.16
*Phaethornis ruber*	a. Be (1) b. N (1)	2.44
*Piculus chrysochloros*	a. Caa (1) b. Ch (1)	2.61
*Piranga flava*	a. Flooded Pm (1) b. Caa (1)	1.98
*Schiffornis turdina*	a. Ron (1) b. N (1)	4.75
*Sittasomus griseicapillus*	a. Caa (1) b.SEAF (2) c. Pu (3)/Ch (3)	3.29
*Terenotriccus erythrurus*	a. Im (1) b. Ron (1)	4.94
*Thalurania furcata*	a. Ron/Pa1 (1) b. Be (1)	2.68
*Thamnomanes caesius*	a. Ron/Pa1 (1) b. N (2) c. Im (1) Gu (1)	6.04
*Thamnophilus aethiops*	a. N (2) b. Ron/Pa1 (2) c. Im (1) d. Be (3)	4.21
*Thryothorus longirostris*	a. Caa (1) b. SEAF (1)	4.6
*Trogon curucui*	a. Caa (1) b. Ron/Pa1 (1)	2.46
*Trogon melanurus*	a. Im (1) b. Pa2 (1)/Ron/Pa1 (1)	2.29
*Xenops minutus*	a. N (1) b. SWAF (1)	5.69
*Xiphorhynchus guttatus*	a. Be (1) b. Ron/N (1)	3.29

a Areas of endemism or ecoregion for each cluster.

b Mmaximum K2P genetic distances within species. Geographic areas coded according Figure 1.

### Phylogeographic patterns

Deep intraspecific divergences in different species were often located between the same pairs of areas of endemism or ecoregions. The most common pattern observed was between the Napo and Rondonian areas of endemism, followed by Belém and Rondonian (Table 4, Figures 4 and 5). However, phylogeographic splits between areas varied in depth in different species. For example, distances between Napo and Rondonian were 8% in *Tolmomyias assimilis*, *Myrmotherula longipennis*, and *Conopophaga aurita*, 5% in *Thamnomanes caesius*, *Cyphorhinus arada*, *Hylophylax naevius*, *Schiffornis turdina*, and *Jacamerops aureus*, and 2% in *Myrmoborus myotherinus*, *Hylophylax poecilinotus*, *Deconychura longicauda*, *Deconychura stictolaema*, *Hemithraupis flavicollis*, *Thamnophilus aethiops*, and *Monasa morphoeus* (Table 4). Between Guyanian and Napo areas of endemism instraspecific divergences of 5 and 2% were observed in *Dendrocincla fuliginosa* and *Jacamerops aureus*, respectively. Conversely, K2P genetic distances were close to zero within the species *Dixiphia pipra* and *Nyctidromus albicollis* (Table 4). Genetic divergences between Belém and Napo were from 2 to 6%, with a wide range of intermediate levels: 2.4 (*Phaethornis ruber*), 2.5 (*Formicarius colma*), 2.8 (*Mionectes oleaginous*), 3.3 (*Xiphorhynchus guttatus*), 3.6 (*Thamnophilus aethiops*), 4.2 (*Jacamerops aureus*), 5.6 (*Dendrocincla fuliginosa*) and 5.7% (*Turdus leucomelas*).

**Table 4:** Most common patterns of geographic distribution in Neotropical bird species surveyed.

Geographic area pairs	Species (K2P distance between locality pair)^a^
1. Napo – Rondonian	*Tolmomyias assimilis* (8.5), *Myrmotherula longipennis* (7.8), *Conopophaga aurita* (7.5), *Thamnomanes caesius* (5.3–5.6), *Cyphorhinus arada* (4.9), *Hylophylax naevius* (4.9), *Schiffornis turdina* (4.8), *Jacamerops aureus* (4.5), *Myrmoborus myotherinus* (2.8), *Hylophylax poecilinotus* (2.5–2.7), *Deconychura longicauda* (2.3), *Deconychura stictolaema* (2.3), *Hemithraupis flavicollis* (2), *Thamnophilus aethiops* (1.8–1.9) *Monasa morphoeus* (1.7)
2. Belem – Napo	*Turdus leucomelas* (5.7–5.9), *Dendrocincla fuliginosa* (5.6), *Jacamerops aureus* (4.2), *Thamnophilus aethiops* (3.6–4.2), *Xiphorhynchus guttatus* (3.3), *Mionectes oleaginous* (2.2–2.8), *Formicarius colma* (2.5), *Phaethornis rubber* (2.4), *Dixiphia pipra* (1.9–2)
3. Belem – Rondonian	*Thamnophilus aethiops* (3.4–4.2), *Malacoptila rufa* (3.6–3.9), *Myrmotherula hauxwelli* (3.4), *Xiphorhynchus guttatus* (3.3), *Thalurania furcata* (2.7), *Jacamerops aureus* (1.9), *Arremon taciturnus* (1.6), *Microcerculus marginatus* (1.6), *Synallaxis rutilans* (1.1–1.2)
4. Guyanian – Napo	*Dendrocincla fuliginosa* (5), *Thamnomanes caesius* (1.8–2.1), *Dixiphia pipra* (0.2), *Nyctidromus albicollis* (0.2)
5. Imerí – Rondonian	*Phaethornis superciliosus* (8.6), *Thamnomanes caesius* (5.8), *Terenotriccus erythrurus* (4.9), *Myrmeciza atrothorax* (3.3), *Hylophylax poecilinotus* (2.6–2.7), *Trogon melanurus* (2.3), *Synallaxis rutilans* (1.5), *Thamnophilus aethiops* (1.8)
6. Atlantic Forest- Caatinga	*Thryothorus longirostris* (4.6), *Crypturellus tataupa* (3.9), *Myiobius atricaudus* (1.7), *Turdus leucomelas* (0.2–0.3)
7. Caatinga- Puna	*Phaeomyias murina* (3–3.2), *Cyclarhis gujanensis* (2.8), *Cyanocompsa brissonii* (2.4–2.7), *Sittasomus griseicapillus* (2.1), *Tyrannus melancholicus* (0–0.6)
8. Caatinga- Chaco	*Cyclarhis gujanensis* (3), *Piculus chrysochloros* (1.8–2.6), *Cyanocompsa brissonii* (0.9–2.5), *Sittasomus griseicapillus* (2.1), *Tyrannus melancholicus* (2.1), *Veniliornis passerinus* (1.8–2)
9. Atlantic Forest- Rondonian/Para 1and 2	*Myiobius barbatus* (12.7), *Turdus albicolis* (4–4.5), *Chloroceryle aenea* (2.2), *Formicivora grisea* (1.6), *Arremon taciturnus* (0.7)

a Corresponding range of K2P genetic distances among samples or clusters from each locality pair.

## Discussion

### Identification of Neotropical species with DNA barcodes

Despite the high success we obtained in Neotropical bird species identification with DNA barcodes (93%), comparable to previous barcode surveys in birds [23], [24], most of the genera and species were not sampled across their entire distribution, which overestimates its potential to differentiate species. This was observed for at least two species previously distinct with DNA barcodes, *Anas sibilatrix*, and *Celeus lugubris* [24], who were shown to be sharing sequences with *Anas flavirostris* and *Celeus elegans*, respectively, when samples from other areas of their geographic range were included in this study. When comprehensive genus and species coverage becomes available in Neotropical birds, more species are likely to not have unique DNA barcodes [21]. Nonetheless, more certainty will be achieved overall in the identification of species with *COI* barcodes because we will be able to better address monophyly of lineages and to verify the frequency with which individuals from different populations within species complexes are exchanging genes [16]. In most of the genera for which we had better species coverage for *COI*, such as *Paroaria*, *Coryphospingus*, *Hemithraupis*, *Cyanerpes*, *Cyanocompsa*, *Mimus*, *Phacellodomus*, and *Dendrocincla*, species did not share barcodes. Even though we obtained only single sequences for many species, they will contribute to future systematic efforts as part of the public standardized DNA barcode library [28]. They also will aid in faster identification of specimens that are difficult to identify morphologically, such as embryos and eggs, which will positively impact the conservation of avian wildlife in the Neotropical region.

### Species not identified by DNA barcodes

Among the species we considered not identifiable with COI barcodes, some were very closely related with very similar barcode sequences (category III, Table 1). Our sampling was not comprehensive enough to reject their monophyly by chance [19], [25], [26], but once more individuals from different areas of their range are included stronger support might be adduced for their reciprocal monophyly [19], [24], [25]. On the other hand some species might be recovered as not monophyletic with increased sample sizes, due to unsorted ancestral polymorphism or hybridization [25]. In that case they would not be identified by DNA barcodes at the species level, suggesting that future studies should employ multilocus phylogenetic inference with faster evolving nuclear sequences in a coalescent framework to try to resolve species lineages [29]. Once larger sample sizes are available for closely related species, character-based approaches implemented automatically, such as in CAOS [30], are preferable to genetic distance levels to determine their distinctiveness, as distance levels within and among species can overlap considerably even when substitutions among species are fully sorted.

The species recovered as non-monophyletic (category IV, Table 1) are strong candidates for taxonomic revision, and some of their divergent lineages might correspond to different species. For instance, the divergent lineages within the bearded flycatcher (*Myiobius barbatus*) belong to different recognized subspecies: *amazonicus*, *insignis*, and *mastacalis* [31], [32]. They currently are allopatric, have morphological differences and differ in their K2P genetic distances by 12.6–13.7%. The three subspecies clades were not recovered as monophyletic with COI barcodes because the ruddy-tailed flycatcher (*Terenotriccus_erythrurus*) and the black-tailed myiobius (*Myiobius_atricaudus*) were included in the species clade. Similarly, specimens from North and South Atlantic forest of the rufous gnateater (*Conopophaga lineata*) differ by 9.6%. However, the lineages from the two localities are not monophyletic because the chestnut-belted gnateater (*Conopophaga aurita*) and the hooded gnateater (*Conopophaga roberti*) are embedded in this group (File S1), as shown previously with more comprehensive sample sizes and mitochondrial markers [33]. The morphological characters used to define these lineages as members of a single species could be under strong stabilizing selection, and thus not mirroring the accumulation of mutations through time in neutral genes like *COI*. Most cases of paraphyly in birds are caused by incorrect taxonomy [34]. Alternatively, paraphyletic species can arise when geographically isolated lineages merge in part of their distribution before complete reproductive isolation has evolved [35]. Phylogeographic studies including samples from their entire geographic range and from the closely related species are needed to properly understand their diversification patterns, and establish their taxonomic status.

The 17 species that shared barcodes with closely related species in sympatry likely experienced hybridization, or recent speciation and incomplete lineage sorting, or could simply be examples of incorrect taxonomy or sample misidentification. For instance, the flightless steamer duck (*Tachyeres pteneres*) shares barcodes with the flying steamer duck (*Tachyeres patachonicus*) in Argentina, even though these species are very distinct morphologically. In this example, misidentification of the sample is less likely. A multigene phylogeny of four duck genera also reported difficulty in resolving the relationships among species of *Tachyeres*, and attributed this to a rapid diversification of the group, with possible incomplete lineage sorting, founder effects, and introgression [36]. The tawny-crowned greenlet (*Hylophilus ochraceiceps*) had intraspecific clusters differing by almost 7% sequence divergence between Napo/Imerí and Rondonian endemic areas, and shared barcodes with the grey-chested greenlet (*H. semecinereus*) in their Rondonian distribution. Both species are comprised of multiple subspecies, and some of their variants are morphologically alike. The current taxonomy of the genus might not be an accurate reflection of lineage relationships, but misidentification of samples cannot be ruled out.

Two species pairs occurring in allopatry were not reciprocally monophyletic: the bicolored (*Gymnopithys leucaspis*) and rufous-throated (*Gymnopithys rufigula*) antbirds, and the ochre-collared piculet (*Picumnus temminckii*) and spotted piculet (*Picumnus pygmaeus*). In both cases they are morphologically distinct and do not share identical barcodes with the other species; genetic distances among samples were around 0.5% and 1.0%, respectively. In these examples the lack of reciprocal monophyly could be result of recent speciation and shared ancestral polymorphism, and hybridization. A faster evolving marker such as the control region or larger mitochondrial sequences might recover their reciprocal monophyly [22], [25].

### Complex patterns of population structure detected with DNA barcodes

Our results agree with previous hypotheses that complex patterns of speciation were responsible for the high diversity in Neotropical bird species [37], and strongly supports the view that most avian species in the region are narrowly endemic rather than widely distributed [9], [38]. Several hypotheses were proposed to explain the patterns of taxon distribution in the Amazonian lowland region. The forest *refugia* hypothesis [5], [39], [40] suggested that cycles of expansion and retraction of dry patches within forest areas were associated with interglacial and glacial periods, and this could create multiple events of isolation among widely distributed groups, promoting speciation [5], [39], [40]. The *riverine* hypothesis suggested that the formation of the rivers in the Amazon region could have acted as important geographic barriers to promote speciation, as they delimit most areas of endemism [41], [42], [43]. This would have started at least by the end of the Miocene with the uplift of the Northern portion of the Andes [44], [45]. Another proposal is the *marine incursions* hypothesis, in which sea-level rises of about 100 m in the Quaternary and late Tertiary are suggested to have fragmented the Amazonian lowland into a large number of true islands and archipelagos, favoring active allopatric speciation [46], [47]. The wide range of divergence levels we observed within the 61 non-monophyletic and monophyletic species with deep intraspecific variation (1–13% K2P distances), together with the high incidence of recently evolved species, is consistent with speciation events starting well before the Pliocene and Pleistocene, and extending to more recent geologic periods [38]. Although several groups of species have similar patterns of genetic and geographic breaks among the same areas of endemism, different levels of genetic distances between the same areas were also recovered in other species. The wide range of intraspecific genetic distances observed between a pair of geographical localities might reflect multiple vicariant events that have occurred at different geological times [15], [48], or they could reflect multiple dispersal events that followed a major isolation process [49], or variation in rates of evolution across different species [50], [51] whose populations were isolated by a single vicariant event. Additionally, a significant relationship was observed in previous studies [52] between interspecific levels of cross-barrier genetic differentiation within the forest stratum at which a species forages in Neotropical rain forest. More comprehensive taxon sampling and estimates of times of diversification that take into account variation in rates of evolution across lineages [50] are needed to properly associate the diversification of a particular taxon with geographical events.

We have chosen not to flag divergent lineages as provisional new species, because our sampling was not comprehensive enough to properly quantify genetic variation in each locality in different species, such as the red-eyed vireo (*Vireo olivaceus*) and the ultramarine grosbeak (*Cyanocompsa brissonii*). Specimens of red-eyed vireo from Puna+Napo and Atlantic Forest were genetically divergent (2–3%), but haplotypes from the Atlantic Forest and Puna were observed in the Chaco. Similarly, specimens of ultramarine grosbeak from Caatinga and Puna were also divergent (2.7%), and both haplotypes are also found in Chaco. Both species may have reinvaded the Chaco after being isolated on the borders of this area. To check if these lineages deserve species recognition it is important to investigate if the highly divergent specimens in sympatric zones are reproductively isolated. Some of the deep intraspecific lineages we described in this study were reported previously, such as the difference among thrush-like Schiffornis (*Schiffornis turdina*) from Rondonian and Napo areas of endemism [11]. Others, such as the whiskered myiobius (*Myiobius barbatus*) from Belém, Para2 and Atlantic forest will likely prove to be different species.

DNA barcodes of several new species of Neotropical birds will contribute to a deeper understanding of the systematics and diversification of these taxa in the area. Assuming the current species taxonomy, studies of historical patterns of diversification of species in the area can be obscured since many species were revealed not to be monophyletic. Moreover, a high number of species in the Neotropical realm are comprised of multiple divergent lineages, thus the sample sizes of barcoded individuals and other markers within and among species in the area need to be higher than in other biogeographic areas that are not as taxon-diverse. This can be achieved by complementary efforts of independent research groups. Common and divergent patterns of genetic distances observed within and among closely related species suggest that multiple geographic processes have shaped the distribution of avian taxa in the Neotropics, and DNA barcodes surveys will continue to reveal many more interesting geographic patterns in the region.

## Materials and Methods

### Taxon sampling

We analyzed 637 individuals from 431 species of Neotropical bird species from two tissue collections: Laboratório de Genética e Evolução Molecular de Aves (LGEMA) in the Universidade de São Paulo, São Paulo, and The Royal Ontario Museum in Toronto (ROM), with high representation in the Amazon lowlands and Atlantic Forest (Table S2, File S2). Whenever available, individuals from different localities of their distribution range were sampled (Table S2, GenBank numbers JN801479 - JN802115, project “Neotropical-BRAS” in the completed projects section of the Barcode of Life Data System- BOLD [53]). To increase intraspecific sampling and to compare more closely related congeneres, we added sequences of individuals from the same species and same genera of Neotropical birds from the study of birds from Argentina [24] (project “Birds of Argentina-Phase I-BARG” in the completed projects section of BOLD [53]), thus extending our survey to 1,431 samples from 561 different species (Table S2, File S2).

### DNA extraction and amplification

DNA was extracted by a membrane purification procedure in glass fiber-filtration plates (Acroprep 96 Filter Plate- 1.0 µm Glass, PALL Corporation) [54], and collected in PCR plates. Sequences of about 700 base pairs (bp) were obtained from the 5′end of the mitochondrial gene Cytochrome oxidase I (*COI*). Polymerase Chain Reaction (PCR) amplifications were performed in 12.5 µL reactions in a buffer solution containing 10 mM Tris-HCl (pH8.3), 50 mM KCl, 2.5 mM MgCl_2_, 0.01% gelatin, 0.4 mM dNTPs, 0.2 µM of each primer, 1 U *Taq* Polymerase (Invitrogen) and 20–25 ng of DNA. Cycle conditions were: an initial denaturation at 94°C for 5 min, 36 cycles of 94°C for 40 sec, 50°C for 40 sec and 72°C for 1 min, and a final extension at 72°C for 7 min. Bird universal primers used in *COI* amplifications were LTyr (forward – TGTAAAAAGGWCTACAGCCTAACGC [19]) and COI907aH2 (GTRGCNGAYGTRAARTATGCTCG [19]) resulting in a long but very stable amplified product of about 910 bp. This primer set successfully amplified the 5′ end of *COI* across a wide range of bird species. The amplified segments were purified by excising bands from agarose gels and centrifuging each through a filter tip [55]. Sequences were obtained on an ABI3730 (*Applied Biosystems*) according to the manufacturers' suggested protocols using the same primer LTyr to sequence the 5′end, and the internal primer COI748Ht (reverse-TGGGARATAATTCCRAAGCCTGG [19]) to sequence the reverse 3′end, resulting in a sequenced product of about 750 bp. Sequences were checked for ambiguities in CodonCode Aligner (*CodonCode Corporation*), and Geneious 5.3 [56].

### Data analyses

Sequences were aligned in Geneious 5.3 using the Geneious alignment algorithm, with gap penalty set as 12.8, and gap extension penalty set as 3. Species and genera counts were performed in the software environment R 2.12 [57]. Genetic distances were calculated under the Kimura 2 –Parameter model (K2P) for all pair-wise comparisons in the matrix using PAUP4b10 [58]. Two datasets of genetic distances were built in R: the first, including all within-species comparisons; and the second, including among-congener comparisons (excluding within-species ones). We wrote R scripts to summarize the mean, variance, maximum, and minimum genetic distances per species and among congeners, respectively, using the first two datasets. Frequency plots of pairwise genetic distances for congeners of different species, and with only within species comparisons were built in R. The maximum likelihood tree topology for the complete dataset was calculated in Geneious 5.3 [56] using PHYML [59]. The best fit-model (General Time Reversible with proportion of invariable sites and gamma, GTR+I+Γ, I = 0.5, Γ = 0.42) was selected with jModelTest [60] with a sample of the original dataset including one or two representative samples of each bird family. Species were considered not distinguishable by DNA barcode if: a) they were not monophyletic; b) they shared barcodes with other species; or c) their intraspecific variation overlapped with the lowest 5% of among-species variation, and reciprocal monophyly of sampled individuals could not be distinguished from random branching at p = 0.05 with the test for chance occurrence of reciprocal monophyly [25], [26].

Within-species clusters with minimum pairwise distances higher than 1.5% K2P were considered for analyses, because this level of genetic distances overlapped with more than 5% of among congeners comparisons (Figure 1), but information on clades differing by less than 1.5% K2P distance is also available (File S1). Species without unique barcodes were sorted into the following non-exclusive categories: I) they share barcodes with species occurring in sympatry or II) they share barcodes with species occurring in allopatry, or III) were monophyletic differing from their sister species by few mutations, or IV) paraphyletic species with lineages more than 1.5% divergent.

For all the paraphyletic and monophyletic species with deep intraspecific divergences, we compared the genetic discontinuities with the geographic locality of the samples. Because areas of endemism are known to harbor unique biota, and many subspecies of birds are delimited also by these zones [38], [39], [40], we classified the sample localities of individuals according to the areas of endemism in the Amazon and in the Atlantic forest where they occur (Figure 1). We adopted the revised areas of endemism in Amazon and Atlantic forest from Bates *et al.* [37] and Borges [61]. Samples collected in other localities were classified according to their respective ecoregion according to the simplified map from Haffer [5] (Figure 2).

## Supporting Information

File S1 **Maximum likelihood tree of 1,431 COI barcodes from the 561 Neotropical bird species surveyed.** Zip file including the tree topology in pdf format. Codes after species names correspond to their Process ID in BOLD (Table S2).
(PDF)

File S2 **Map representing the sample distribution in the Neotropical region.** Blue dots correspond to new samples sequenced for this study (BOLD project BRAS), and red dots correspond to samples available from literature (BOLD project BARG). Dots may correspond to the locality of multiple samples.
(TIF)

Table S1 **Within- and amongspecies mean, variance, minimum, and maximum K2P genetic distances.**
(XLS)

Table S2 **Specimen details.** List of specimens used in the study, with detailed identification information, and sample locality coordinates (excel file).
(XLS)

## References

[1] Gaston K (2000). Global patterns in biodiversity.. Nature.

[2] Cardillo M, Orme CDL, Owens IPF (2005). Testing for latitudinal bias in diversification rates: an example using new world birds.. Ecology.

[3] Hawkins BA, Diniz-Filho JAF (2004). ‘Latitude’ and geographic patterns.. Ecography.

[4] Rohde K (1999). Latitudinal gradients in species diversity and Rapoport's rule revisited: a review of recent work and what can parasites teach us about the causes of the gradients?. Ecography.

[5] Haffer J (1985). Avian zoogeography of the Neotropical lowlands.. Ornithol Monogr.

[6] Price T (2008). Speciation in Birds.

[7] Ribas CC, Miyaki CY (2004). Molecular systematics in *Aratinga* parakeets: species limits and historical biogeography in the ‘*solstitialis*’ group, and the systematic position of *Nandayus nenday*.. Mol Phylogenet Evol.

[8] Carantón-Ayala D (2010). A new species of antpitta (Grallariidae: Grallaria) from the northern sector of the western Andes of Colombia.. Ornitol Colomb.

[9] Cheviron ZA, Hackett SJ, Capparella AP (2005). Complex evolutionary history of a Neotropical lowland forest bird (*Lepidothrix coronata*) and its implications for historical hypotheses of the origin of Neotropical avian diversity.. Mol Phylogenet Evol.

[10] Marks BD, Hackett SJ, Capparella AP (2002). Historical relationships among Neotropical lowland forest areas of endemism as determined by mitochondrial DNA sequence variation within the Wedge-billed Woodcreeper (Aves: endrocolaptidae: *Glyphorynchus spirurus*).. Mol Phylogenet Evol.

[11] Nyári AS (2007). Phylogeographic patterns, molecular and vocal differentiation, and species limits in *Schiffornis turdina* (Aves).. Mol Phylogenet Evol.

[12] Ribas CC, Tavares ES, Yoshihara C, Miyaki CY (2007). Phylogeny and biogeography of Yellow-headed and Blue-fronted Parrots (*Amazona ochrocephala* and *Amazona aestiva*) with special reference to the South American clade.. Ibis.

[13] Joseph L, Wilke T, Bermingham E, Alpers D, Ricklefs R (2004). Towards a phylogenetic framework for the evolution of shakes, rattles, and rolls in Myiarchus Tyrant-flycatchers (Aves: Passeriformes: Tyrannidae).. Mol Phylogenet Evol.

[14] Amaral FS, Miller MJ, Silveira LF, Bermingham E, Wajntal A (2006). Polyphyly of the hawk genera *Leucopternis* and *Buteogallus* (Aves, Accipitridae): multiple habitat shifts during the Neotropical buteonine diversification.. BMC Evol Biol.

[15] Miller MJ, Bermingham E, Klicka J, Escalante P, do Amaral FS (2008). Out of Amazonia again and again: episodic crossing of the Andes promotes diversification in a lowland forest flycatcher.. Proc Biol Sci.

[16] Hebert PD, Cywinska A, Ball SL, deWaard JR (2003). Biological identifications through DNA barcodes.. Proc Biol Sci.

[17] Kerr KCR, Stoeckle MY, Dove CJ, Weigt LA, Francis CM (2007). Comprehensive DNA barcode coverage of North American birds.. Mol Ecol Notes.

[18] Hebert PD, Stoeckle MY, Zemlak TS, Francis CM (2004). Identification of Birds through DNA Barcodes.. PLoS Biol.

[19] Tavares ES, Baker AJ (2008). Single mitochondrial gene barcodes reliably identify sister-species in diverse clades of birds.. BMC Evol Biol.

[20] Yoo HS, Eah JY, Kim JS, Min MS, Paek WK (2006). DNA barcoding Korean birds.. Mol Cell.

[21] Moritz C, Cicero C (2004). DNA barcoding: promise and pitfalls.. PLoS Biol.

[22] Milá B, Girman DJ, Kimura M, Smith TB (2000). Genetic evidence for the effect of a postglacial population expansion on the phylogeography of a North American songbird.. Proc R Soc Lond Biol Sci.

[23] Vilaça ST, Lacerda DR, Sari EHR, Santos FR (2006). DNA-based identification applied to Thamnophilidae (Passeriformes) species: the first barcodes of Neotropical birds.. Rev Bras Ornitol.

[24] Kerr KCR, Lijtmaer DA, Barreira AS, Hebert PDN, Tubaro PL (2009). Probing evolutionary patterns in Neotropical birds through DNA barcodes.. PLoS ONE.

[25] Baker AJ, Tavares ES, Elbourne R (2009). Countering criticisms of single mitochondrial DNA gene barcoding in birds.. Mol Ecol Res.

[26] Rosenberg NA (2007). Statistical tests for taxonomic distinctiveness from observations of monophyly.. Evolution.

[27] Sigrist T (2009). The Avis Brasilis Field Guide to the Birds of Brazil.

[28] Ratnasingham S, Hebert PDN (2007). BOLD: The Barcode of Life Data System.. Mol Ecol Notes.

[29] Heled J, Drummond AJ (2010). Bayesian inference of species trees from multilocus data.. Mol Biol Evol.

[30] Rach J, DeSalle R, Sarkar IN, Schierwater B, Hadrys H (2008). Character-based DNA barcoding allows discrimination of genera, species and populations in Odonata.. Proc R Soc Lond Biol Sci.

[31] Fitzpatrick JW, Bates JM, Bostwick KS, Caballero IC, Clock BM, Del Hoyo J, Elliot A, Christie D (2004). Family Tyrannidae (Tyrant-flycatchers).. Handbook of the Birds of the World Volume 9: Cotingas to Pipits and Wagtails.

[32] Integrated Taxonomic Information System (http://www.itis.gov/index.html)

[33] Pessoa RO (2008). Sistemática e Biogeografia Histórica da Família Conopophagidae (Aves: Passeriformes): Especiação nas Florestas da América do Sul [PhD].

[34] McKay BD, Zink RM (2010). The causes of mitochondrial DNA gene tree paraphyly in birds.. Mol Phylogenet Evol.

[35] de Queiroz K (2005). A unified concept of species and its consequences for the future of taxonomy.. Proc Calif Acad Sci.

[36] Bulgarella M, Sorenson MD, Peters JL, Wilson RE, McCracken KG (2010). Phylogenetic relationships of *Amazonetta*, *Speculanas*, *Lophonetta*, and *Tachyeres*: four morphologically divergent duck genera endemic to South America.. J Avian Biol.

[37] Bates JM, Hackett SJ, Cracraft J (1998). Area-relationships in the Neotropical lowlands: an hypothesis based on raw distributions of Passerine birds.. J Biogeogr.

[38] Cracraft J (1985). Historical biogeography and patterns of differentiation within the South American avifauna: areas of endemisms.. Ornithol Monog.

[39] Haffer J (1969). Speciation in Amazonian forest birds.. Science.

[40] Haffer J (1974).

[41] Ayres M, Clutton-Brock TH (1992). River boundaries and species range size in Amazonian primates.. Am Nat.

[42] Haffer J (1992). On the “river effect” in some forest birds of southern Amazonia.. Bol Mus Para Emilio Goeldi Ser Zool.

[43] Sick H, Lent H (1967). Rios e enchentes na Amazonia como obstáculo para a avifauna.. Atlas do Simpósio sobre a Biota Amazonica- Zoologia.

[44] Hoorn C (1994). An environmental reconstruction of the palaeo-Amazon River system (Middle-Late Miocene, NW Amazonia).. Palaeogeogr Palaeoclimatol Palaeoecol.

[45] Hoorn C, Guerrero J, Sarmiento GA, Lorente MA (1995). Andean tectonics as a cause for changing drainage patterns in Miocene northern South America.. Geology.

[46] Nores M (1999). An alternative hypothesis for the origin of Amazonian bird diversity.. J Biogeogr.

[47] Marroig G, Cerqueira R (1997). Plio-Pleistocene South American history and the Amazon laggon hypothesis: a piece in the puzzle of amazonian diversification.. J Comp Biol.

[48] Brumfield RT, Edwards SV (2007). Evolution into and out of the Andes: a Bayesian analysis of historical diversification in *Thamnophilus antshrikes*.. Evolution.

[49] Leache AD, Crews SC, Hickerson MJ (2007). Two waves of diversification in mammals and reptiles of Baja California revealed by hierarchical Bayesian analysis.. Biol Lett.

[50] Pereira SL, Baker AJ (2006). A mitogenomics timescale for birds detects variable phylogenetic rates of molecular evolution and refutes the standard molecular clock.. Mol Biol Evol.

[51] Nabholz B, Glemin S, Galtier N (2008). Strong variations of mitochondrial mutation rate across mammals: the longevity hypothesis.. Mol Biol Evol.

[52] Burney CW, Brumfield RT (2009). Ecology predicts levels of genetic differentiation in Neotropical birds.. Am Nat.

[53] Barcode of Life Data System (http://www.barcodinglife.org)

[54] Ivanova NV, DeWaard JR, Hebert PDN (2006). An inexpensive, automation-friendly protocol for recovering high-quality DNA.. Mol Ecol Notes.

[55] Dean AD, Greenwald JE (1995). Use of filtered pipet tips to elute DNA from agarose gels.. Biotechniques.

[56] Drummond AJ, Ashton B, Buxton S, Cheung M, Cooper A (2010). http://www.geneious.com.

[57] R Development Core Team (2010). R: A language and environment for statistical computing. 2.12 ed.

[58] Swofford DL (2002). PAUP*: Phylogenetic Analysis Using Parsimony (*and related methods) 4ed.

[59] Guindon S, Gascuel O (2003). A simple, fast, and accurate algorithm to estimate large phylogenies by maximum likelihood.. Syst Biol.

[60] Posada D (2008). jModelTest: Phylogenetic Model Averaging.. Mol Biol Evol.

[61] Borges SH (2007). Análise biogeográfica da avifauna da região oeste do baixo Rio Negro, amazônia brasileira.. Rev Bras Zool.

[62] Campagna L, Lijtmaer DA, Kerr KCR, Barreira AS, Hebert PD (2010). DNAbarcodes provide new evidence of a recent radiation in the genus *Sporophila* (Aves: Passeriformes).. Mol Ecol Res.

