# Supplementary figures and images for: DNA Barcode Detects High Genetic Structure within Neotropical Bird Species
Source: PLoS One. 2011 Dec 7;6(12):e28543. doi: 10.1371/journal.pone.0028543 (PMC3233584; doi:10.1371/journal.pone.0028543)

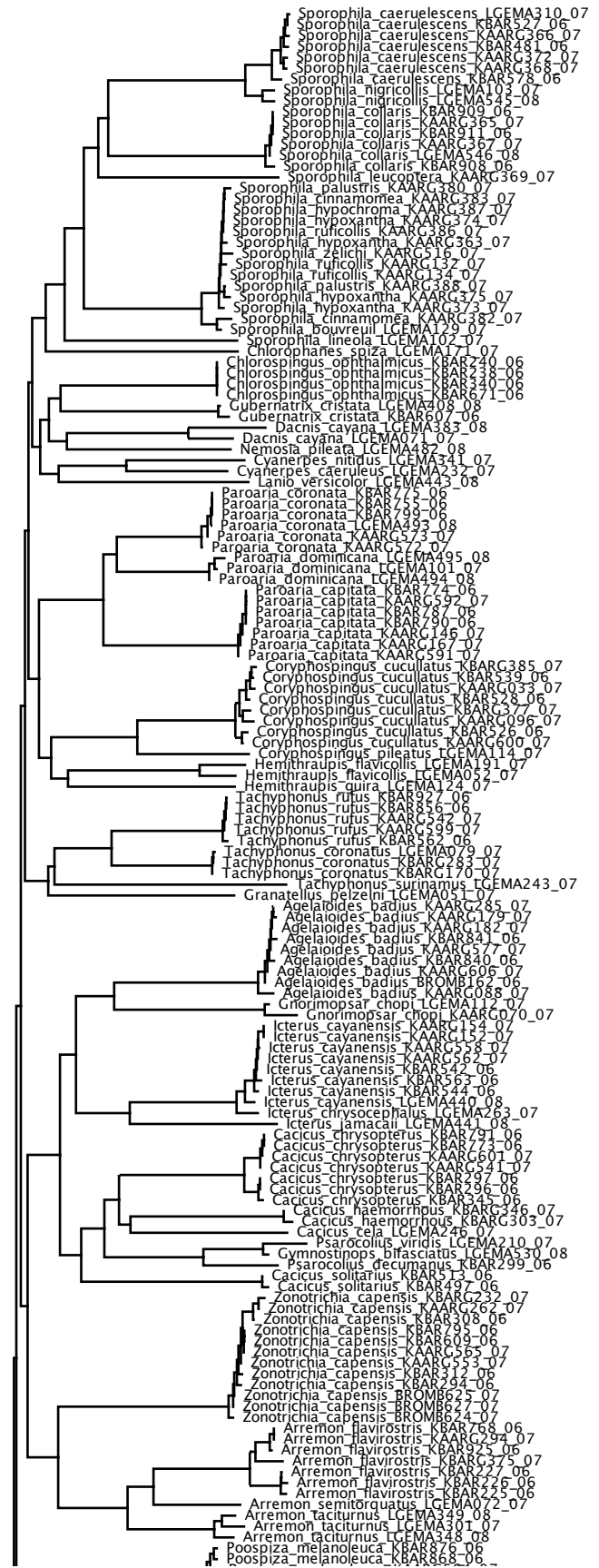

Page 1 of 10.

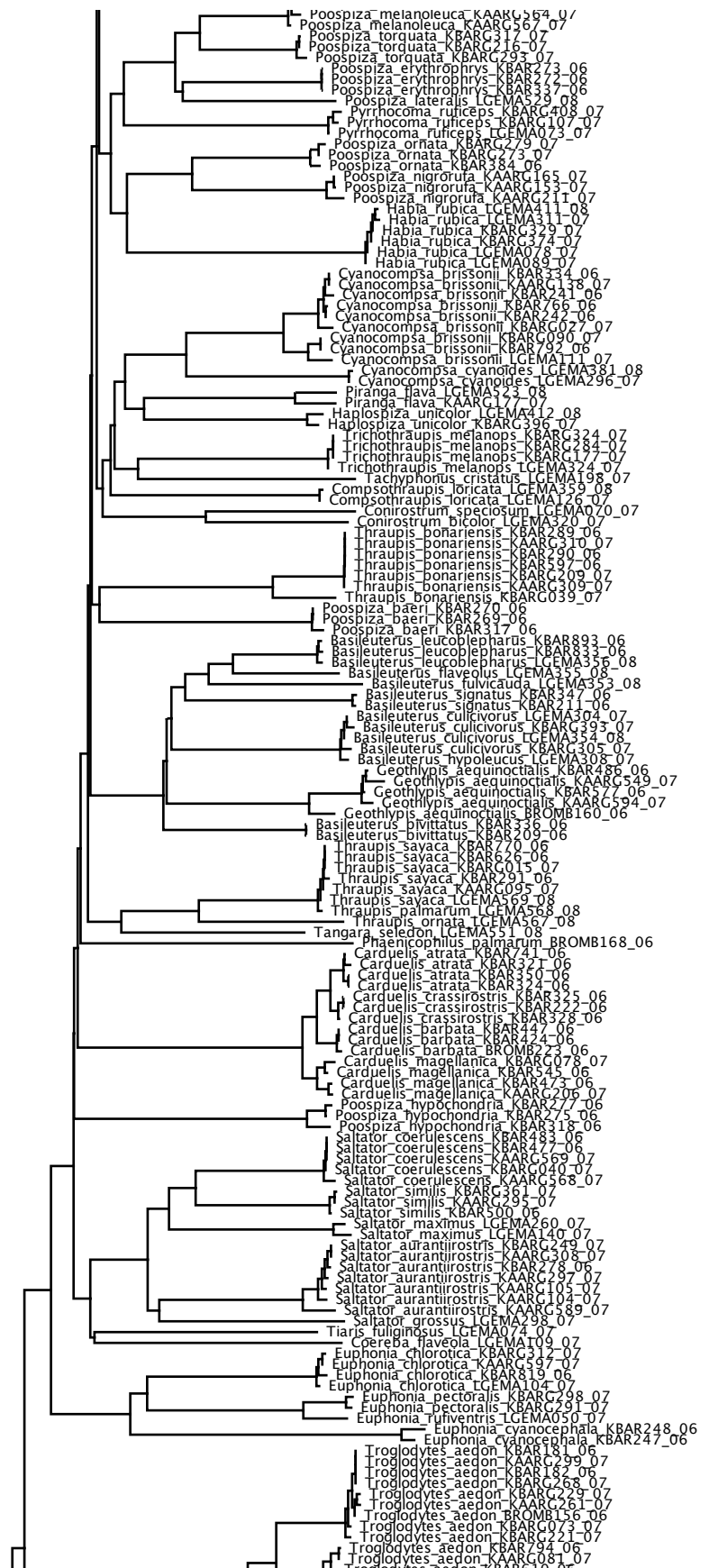

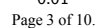

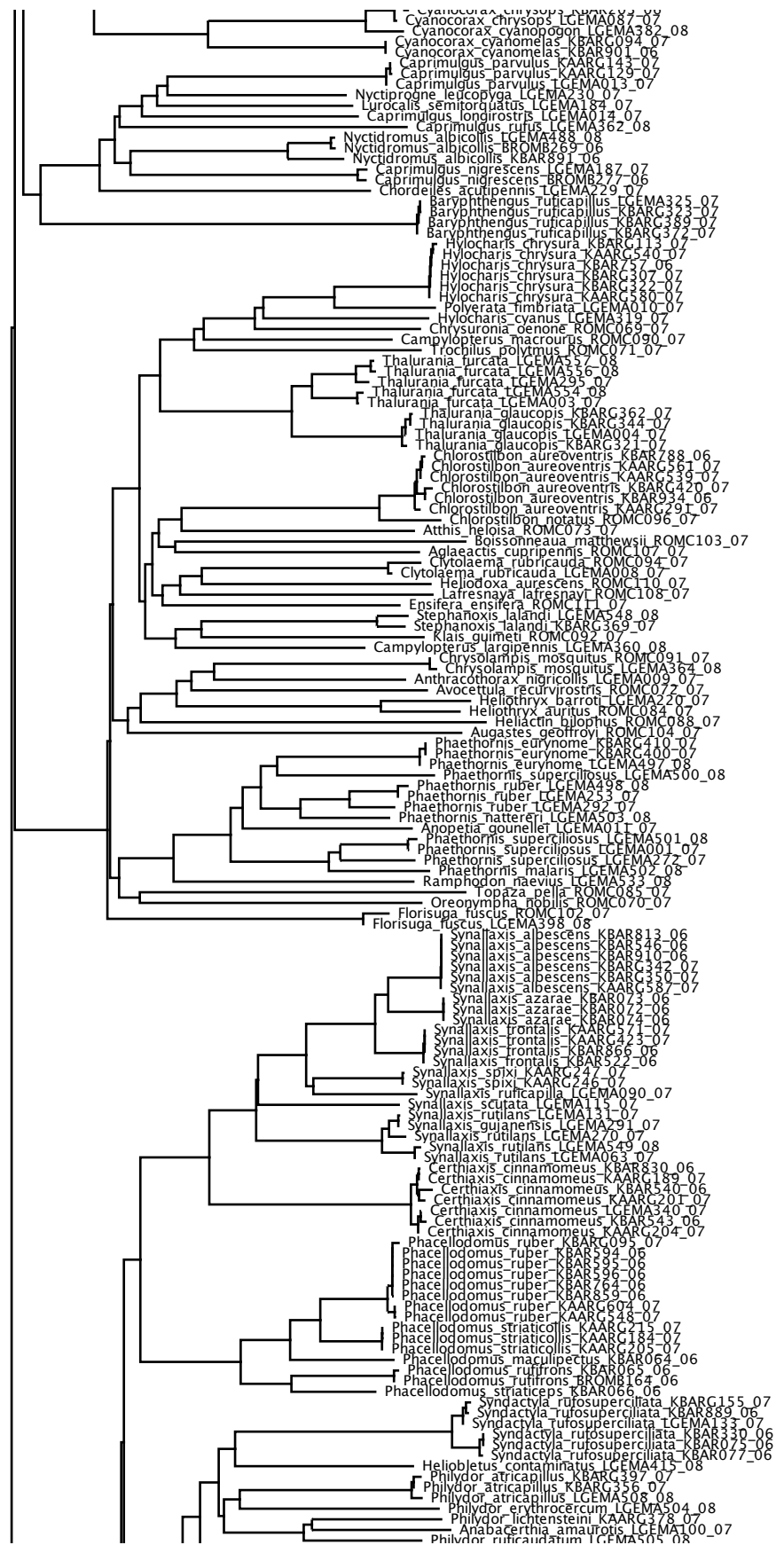

0.01

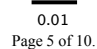

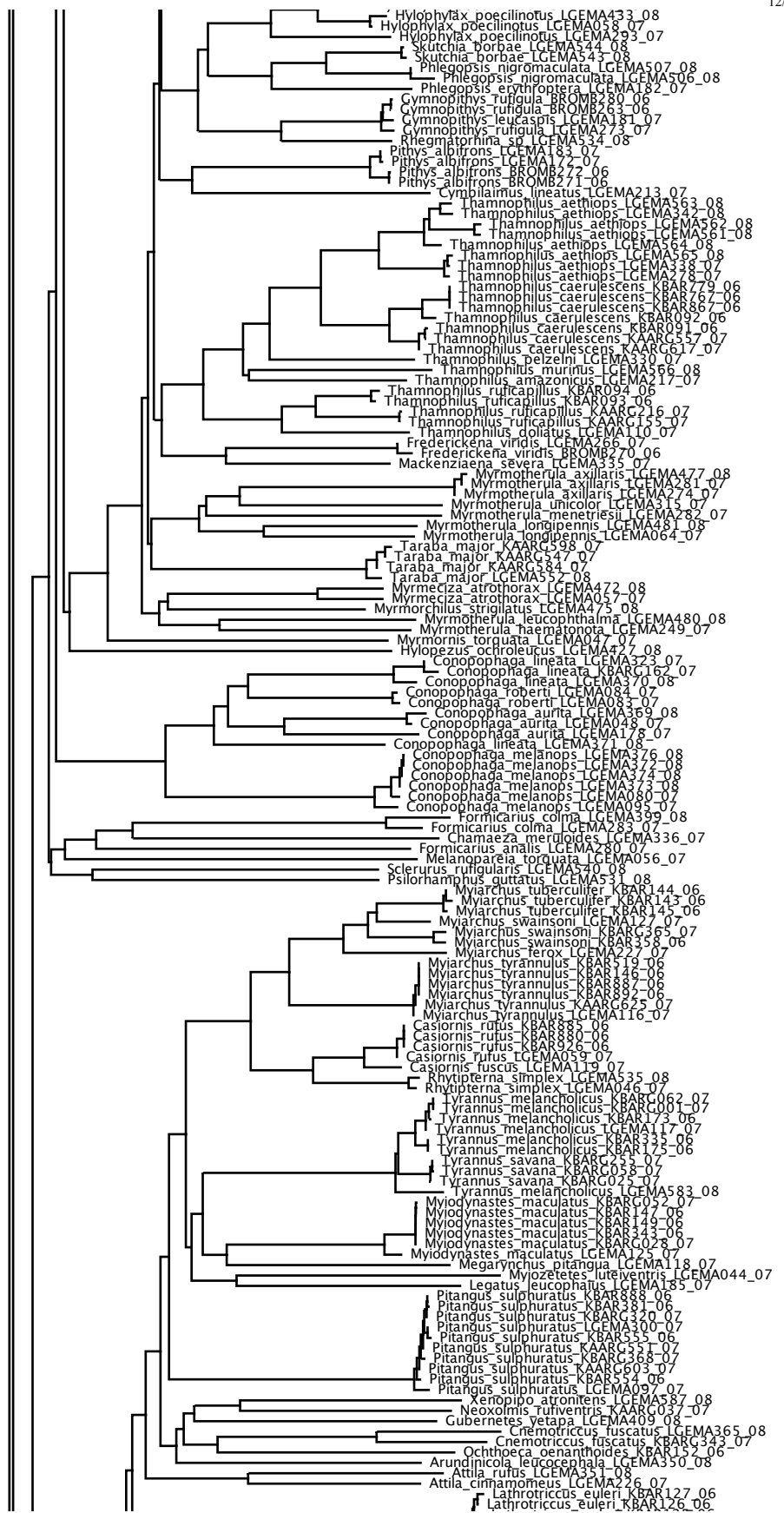

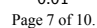

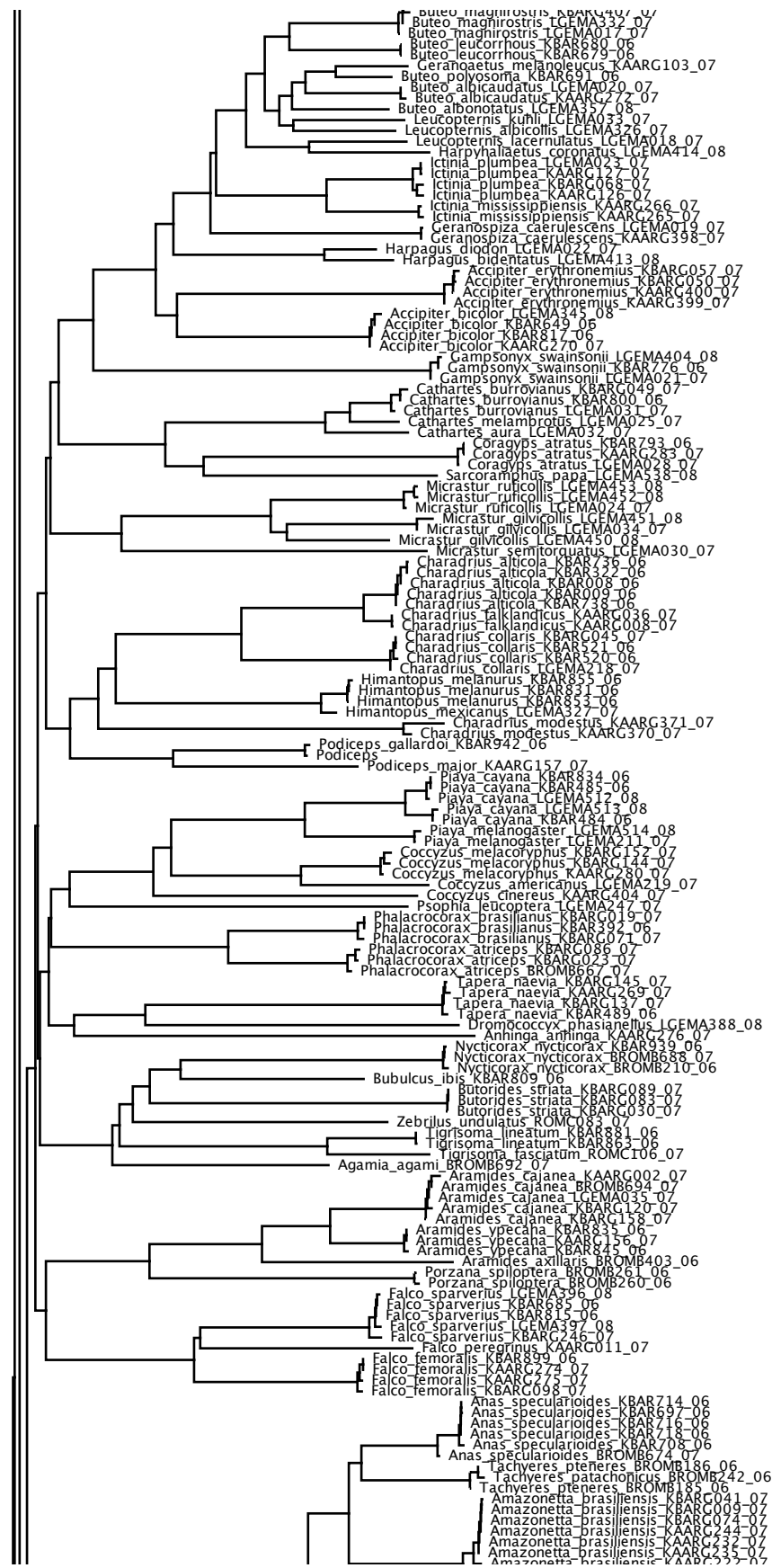

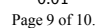

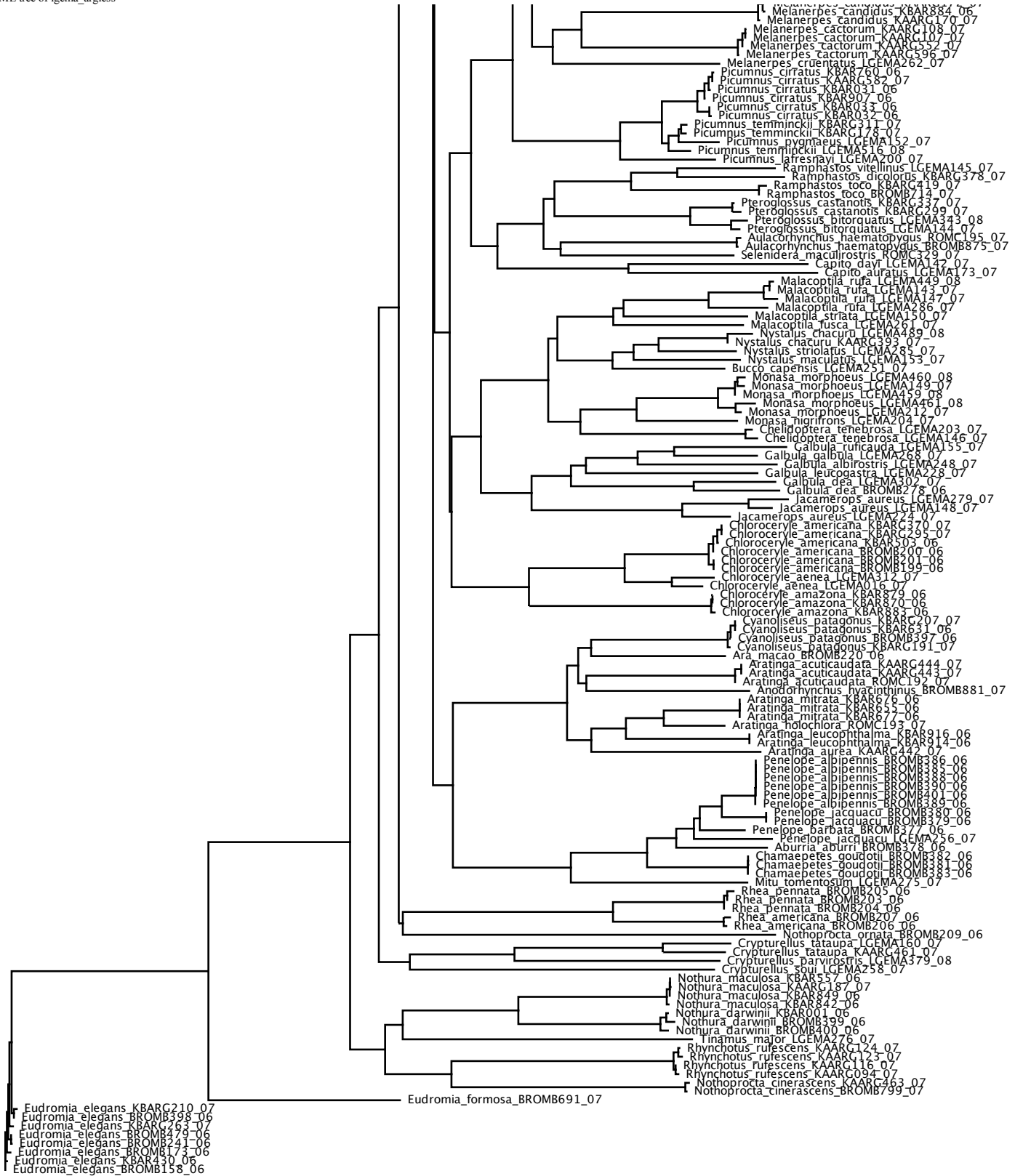

Supplement: File S1 — Maximum likelihood tree of 1,431 COI barcodes from the 561 Neotropical bird species surveyed. Zip file including the tree topology in pdf format. Codes after species names correspond to their Process ID in BOLD (Table S2). (PDF) [file pone.0028543.s001.pdf]

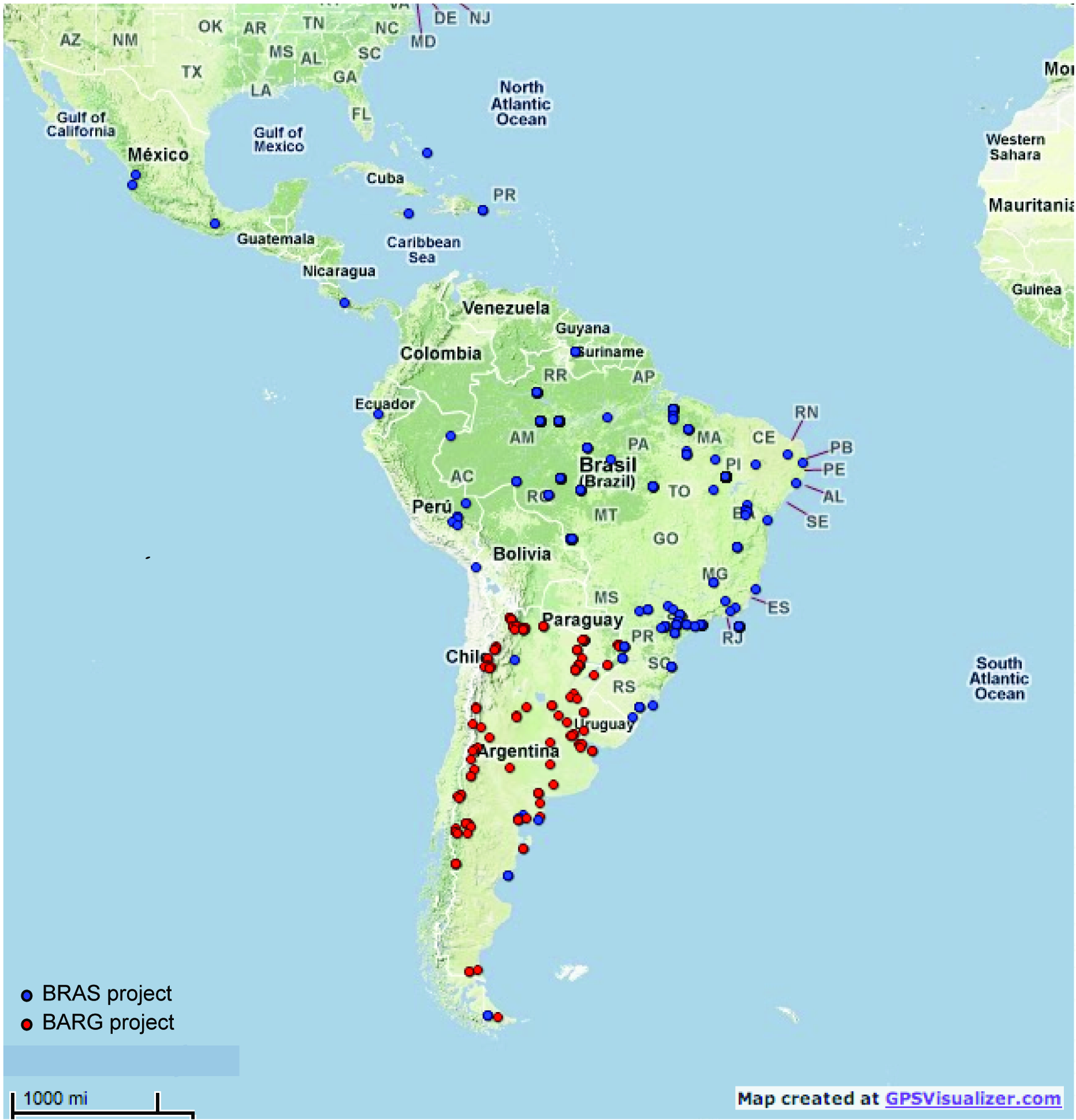

Supplement: File S2 — Map representing the sample distribution in the Neotropical region. Blue dots correspond to new samples sequenced for this study (BOLD project BRAS), and red dots correspond to samples available from literature (BOLD project BARG). Dots may correspond to the locality of multiple samples. (TIF) [file pone.0028543.s002.tif]
